# The Heterogeneity, Distribution, and Environmental Associations of *Borrelia burgdorferi* Sensu Lato, the Agent of Lyme Borreliosis, in Scotland

**DOI:** 10.3389/fpubh.2014.00129

**Published:** 2014-08-28

**Authors:** Marianne C. James, Lucy Gilbert, Alan S. Bowman, Ken J. Forbes

**Affiliations:** ^1^Institute of Biological and Environmental Sciences, University of Aberdeen, Aberdeen, UK; ^2^Division of Applied Medicine, University of Aberdeen, Aberdeen, UK; ^3^James Hutton Institute, Aberdeen, UK

**Keywords:** *Ixodes ricinus*, ticks, Lyme disease, MLST, PCR, genetics, allele, sequence type

## Abstract

Lyme borreliosis is an emerging infectious human disease caused by the *Borrelia burgdorferi* sensu lato complex of bacteria with reported cases increasing in many areas of Europe and North America. To understand the drivers of disease risk and the distribution of symptoms, which may improve mitigation and diagnostics, here we characterize the genetics, distribution, and environmental associations of *B. burgdorferi* s.l. genospecies across Scotland. In Scotland, reported Lyme borreliosis cases have increased almost 10-fold since 2000 but the distribution of *B. burgdorferi* s.l. is so far unstudied. Using a large survey of over 2200 *Ixodes ricinus* tick samples collected from birds, mammals, and vegetation across 25 sites we identified four genospecies: *Borrelia afzelii* (48%), *Borrelia garinii* (36%), *Borrelia valaisiana* (8%), and *B. burgdorferi* sensu stricto (7%), and one mixed genospecies infection. Surprisingly, 90% of the sequence types were novel and, importantly, up to 14% of samples were mixed intra-genospecies co-infections, suggesting tick co-feeding, feeding on multiple hosts, or multiple infections in hosts. *B. garinii* (hosted by birds) was considerably more genetically diverse than *B. afzelii* (hosted by small mammals), as predicted since there are more species of birds than small mammals and birds can import strains from mainland Europe. Higher proportions of samples contained *B. garinii* and *B. valaisiana* in the west, while *B. afzelii* and *B. garinii* were significantly more associated with mixed/deciduous than with coniferous woodlands. This may relate to the abundance of transmission hosts in different regions and habitats. These data on the genetic heterogeneity within and between *Borrelia* genospecies are a first step to understand pathogen spread and could help explain the distribution of patient symptoms, which may aid local diagnosis. Understanding the environmental associations of the pathogens is critical for rational policy making for disease risk mitigation and land management.

## Introduction

Lyme borreliosis is the most prevalent tick-borne human disease in the northern hemisphere, and is growing in incidence in Europe. For example, Scotland has seen an almost 10-fold increase in reported cases since 2000 (1). The causative agent of Lyme borreliosis is *Borrelia burgdorferi* sensu lato, a complex of related spirochete bacteria comprising a suite of genospecies, which vary in pathogenicity and cause different symptoms. The global distribution of the genospecies differs between continents, for example, *Borrelia afzelii* and *Borrelia garinii* are found only in Europe, *Borrelia carolinensis* is found only in North America, and *B. burgdorferi* sensu stricto is found on both sides of the Atlantic (2).

There are currently at least 18 proposed and confirmed *B. burgdorferi* genospecies globally, which vary in their pathogenicity, reservoir host associations, and geographic distributions within and between countries (3–5). Three genospecies (*B. burgdorferi* s.s., *B. garinii*, and *B. afzelii*) are commonly reported to cause Lyme borreliosis and *B. valaisiana* and *Borrelia lusitaniae* may also be pathogenic (6–10).

*Borrelia burgdorferi* s.l. is transmitted by *Ixodes* ticks and, in most of Europe, including the UK, the principle vector is *Ixodes ricinus*. *I. ricinus* are generalist ecto-parasites, feeding on most terrestrial vertebrate species. However, each *B. burgdorferi* s.l. genospecies is specialized and associated with a particular host type. *B. garinii* and *B. valaisiana* are commonly found in birds, *B. afzelii* is associated with small mammals, and *B. burgdorferi* s.s. is associated with both birds and small mammals (11–13). We may therefore predict that genospecies prevalence varies with the relative abundance of these host types or, as a proxy, with different habitats that are associated with these hosts. In addition, within host types, some species are more effective at pathogen transmission than others, so we might predict within-genospecies genetic diversity to vary, for example, we might predict that *B. garinii* may have higher genetic diversity than *B. afzelii* because there are many more species of birds than small mammals. In addition, if genetic variation over space is driven by host movements [e.g., Ref. (14, 15)], we can predict that sequence types within genospecies are more closely related if they occur in closer geographic proximity; we can use spatial genetic variation to infer host movement behavior and in turn this helps us identify how each pathogen spreads over space.

*Ixodes ricinus* has three active stadia (larvae, nymphs, and adults), each of which requires a single blood meal. Unfed larvae are almost always uninfected because vertical transmission of *B. burgdorferi* s.l. from adult females to larvae is extremely rare (16). Despite the host specificity of each genospecies of *B. burgdorferi* s.l. and the general assumption that each tick stage feeds on only one host, co-infections of both “bird” and “mammal” genospecies can occur within a tick (3), although the frequency of such co-infections is less than expected (17). Such co-infections might suggest that the host specificity of genospecies is not absolute, or a tick stage may occasionally take more than one blood meal (from more than one host type), or through co-feeding [transmission from one tick to another without systemic host infection (18, 19)]. This interesting phenomenon warrants further research and one of our aims is to quantify the frequency and type of mixed infections in our studied *I. ricinus* populations.

The molecular characterization of *B. burgdorferi* genospecies and strains has been revolutionized by multi-loci sequence typing (MLST) and in this study we use the system developed by Margos and others (20) which has been shown to unambiguously delineate genospecies and establish evolutionary and geographic relationships. DNA can be directly amplified by polymerase chain reaction (PCR) from tick extracts and the amplicons sequenced without need for culture. The *B. burgdorferi* MLST website^1^ currently documents more than 1500 *B. burgdorferi* strains in the MLST database, currently comprising 572 sequence types from sites in Europe, North America, and Asia.

Previous MLST of studies of *B. burgdorferi* s.l. have been conducted at a continental scale (15, 20, 21) or focused on a single genospecies (20, 22, 23). Our study differs by employing a dense stratified survey to genetically and ecologically characterize the full suite of genospecies at a national scale. Characterizing the variety, distribution, and abundance of sequence types (i.e., the allelic profile) of *B. burgdorferi* s.l. in one country, especially linked to environmental information, will contribute to our understanding of the heterogeneous distribution and prevalence of genospecies, how the pathogens spread, identify the environmental risk factors and will also have implications for patient symptoms and diagnosis. We focused our *B. burgdorferi* s.l. survey on Scotland, where reported cases of Lyme borreliosis have increased almost 10-fold since the turn of the millenium, and where there have been very few previous studies (and no large-scale systematic surveys) of *B. burgdorferi* s.l. genospecies; (24) reported five *B. afzelii* samples and seven *B. burgdorferi* s.s. while (15) genotyped three *B. burgdorferi* samples from one Scottish site and found all to be *B. afzelii*.

This study therefore aimed to examine the phylogenetic population structure of Scottish *B. burgdorferi* s.l. by characterizing the genospecies, sequences types, and alleles and describing their spatial distribution across the country and identifying environmental and regional associations. This was to provide the first large-scale fundamental data on the Lyme borreliosis agents across this country and to gain insight into genetic mixing spatially across Scotland and with other countries (e.g., due to host movements). Furthermore, we were particularly interested in identifying mixed infections (both between genospecies and between sequence types within genospecies) within individual ticks, since this has implications both for patient symptoms and diagnosis and for our understanding of tick feeding behavior. We also aimed to correlate *B. burgdorferi* s.l. genospecies with environmental factors, which is of use in understanding the relationship between host communities and genospecies composition, and in assessing disease risk and mitigation options in different environments.

## Materials and Methods

### Field collection of ticks

Of the three life stages, nymphs are thought to pose the greatest risk to humans in terms of transmitting *B. burgdorferi* s.l.: questing larvae very rarely carry the pathogens (16), while adults are much less numerous than nymphs and are much larger and therefore more likely to be noticed and removed quickly. Indeed, (25) estimated that 82% of human tick bites from a forested area in England were from nymphs. Therefore, this study concentrated primarily on *I. ricinus* nymphs. Questing (host-seeking) nymphs were collected during blanket dragging surveys at 25 woodland sites across Scotland in the springs and summers of 2007–2008 [see Ref. (26)]. A 1 m × 1 m square of blanket material was dragged for 10 m and all ticks counted and collected. At least 20 drags were conducted at each site in a semi-random fashion so as to cover the site in a representative way (26). At least 50 nymphs per site were screened for *B. burgdorferi* s.l. (see method below) including at least one nymph from each drag. Woodland was chosen because it is the habitat most often associated with high densities of a variety of tick species, both in Europe and North America [e.g., Ref. (27–30)] and the habitat most associated with acquiring Lyme borreliosis in Scotland (31). A broad geographic spread of sites ensured as much coverage over the country as possible, while collecting ticks from both semi-natural mixed/deciduous and conifer forests ensured that the main woodland habitat types (and therefore by proxy a range of host communities) were sampled. In addition, each site was associated with known cases of Lyme borreliosis (31).

As well as sampling questing nymphs from the 25 woodland sites, ticks were also removed from hosts. Passerine birds were trapped at one of the woodland sites by mist netting, under license issued by the British Trust for Ornithology during the spring and summer of 2008 [see Ref. (32)]. Feeding nymphs and larvae was removed from birds and stored in vials of 70% ethanol. Small mammals (wood mice *Apodemus sylvaticus* and bank voles *Myodes glareolus*) were trapped using longworth live traps, under license from Scottish Natural Heritage, at four of the woodland sites during the spring and summer of 2007. Ticks were removed and stored in vials per animal in 70% ethanol.

### Tick pooling for analysis

Questing *I. ricinus* nymphs collected in 2007 were each homogenized and amplified individually by PCR. However, questing nymphs from 2008 were pooled in groups of five for processing. Our screening of nymphs collected in 2007 determined that the mean infection prevalence of *B. burgdorferi* s.l. was around 5%, therefore, the probability of more than one positive tick occurring in a pool of five was only 2.3%. Pooling was undertaken only for ticks collected at the same site on the same visit (and in the majority of cases from the same 10 m × 1 m survey transect).

Feeding nymphal ticks removed from birds were PCR amplified individually (as feeding nymphs have now fed on two hosts). Feeding larval ticks removed from small mammals were pooled per animal. This was because transovarial transmission is thought to occur at very low frequencies (16) such that any larval ticks collected from one animal should have been exposed to *B. burgdorferi* s.l. present in that particular animal only. Between 2 and 28 larvae were removed from each small mammal and pooled for PCR (nymph ticks from mammals were collected in very small numbers and not assayed).

### *Borrelia burgdorferi* s.l. screening and MLST

DNA extraction was performed by mechanical destruction of the nymph and larval ticks by ammonia extraction (32, 33). A nested PCR for the 5S-23S rRNA IGS and visualization by agar electrophoresis were used to detect *B. burgdorferi* s. l. (34). A positive control of *B. lusitaniae* (a genospecies not found in northern Europe) and a negative control were used in all PCRs so that it was possible to detect any cross contamination or false positives. MLST, which has been shown to unambiguously delineate genospecies (20), was used to type all positive samples at eight loci (*clpA, clpX, pepX, pyrG, recG, rplB*, and *uvrA*) (after 20). Positive controls were also typed and comprised *B. afzelii* (strain VS461), *B. garinii* (20047), *B. valaisiana* (VS116), *B. burgdorferi* s.s. (B31), and *B. lusitaniae* (Poti B2). PCR products were sequenced in both directions using an ABI automated DNA sequencer.

Forward and reverse sequences were compared, aligned, and trimmed using Sequence Editor (version 1.0.3, Macintosh computers) and consensus sequences were assigned an allele number. New alleles were submitted to the MLST website^1^.

### Phylogenetic analysis

Phylogenetic trees were drawn using MEGA [Version 4.0.2 (35)], which was also used to calculate pair-wise genetic distances and nucleotide differences between sequences. We also used this analysis to test our prediction that *B. garinii* should have greater genetic diversity within Scotland than *B. afzelii* [because there are more birds than small mammal species in Scotland (and Europe) and birds can carry mainland European strains into Scotland].

To test our prediction that samples should be more genetically similar if they are from sites in close proximity (and genetically dissimilar if they are far apart), we used linear regression analysis to examine the relationship between the geographical distance (kilometer) between the collection sites and the molecular diversity in *B. afzelii* and *B. garinii* samples [as defined by the number of single nucleotide polymorphism differences, calculated in MEGA version 4.0.2 (35)]. We compared models that were fit using distance (distance)^2^, square root (distance), and log (distance + 1) in order to test for both linear and curvilinear relationships. We chose the best fit based on model outputs of *R*-squared and *F* values and residual fits.

To estimate whether we sampled enough ticks to provide a full picture of alleles and sequence types over Scotland we used rarefaction curves. This is a standard method to gage the extent to which sampling achieves saturation coverage (i.e., all types of individual or species in a population are sampled) (36). By plotting the number of samples analyzed against the number of sequence types and alleles found for each of the eight loci, the shape of the curve indicates whether most of the alleles or sequence types have been found (i.e., when the curve plateaus because few additional alleles are being found) or whether there are many more to be discovered (i.e., the curve is still climbing steeply because more alleles are being found). Rarefaction curves were drawn in Analytical Rarefaction 1.3 (University of Georgia), both with data from this study and with *B. burgdorferi* s.s. data taken from Ref. (20).

### Identifying mixed infections

To investigate which novel sequence types found may not be real sequence types but, instead, may be a result of mixed allele infections, we closely examined each allele combination in relation to sequence type using only those samples that were successfully analyzed at eight loci (Table S1 and Figure S1 in Supplementary Material). It was assumed that sequence types that are represented by more than one sample or have previously been added to pubMLST are genuine sequence types. It was also assumed that single locus variants of a sequence type with more than one occurrence were also genuine sequence types. In order to estimate the frequency of intra-genospecies co-infections, we examined *B. afzelii* genotypes because *B. afzelii* was the most frequently found genospecies (and, as the results show, had a very large proportion of novel sequence types).

As well as mixed infections, there are alternative hypotheses for novel and single-occurring sequence types. For example, they could arise as the result of homoplasy (the independent acquisition of the same nucleotide polymorphism in an unrelated lineage due to mutation) or horizontal gene transfer (recombination). We considered homoplasy to be unlikely if there was more than one single nucleotide polymorphism. We tested for horizontal gene transfer by using Clonal Frame (version 1.1) to examine the clonal relationships between sequence types and to estimate the recombination events, which may have disrupted inheritance (37).

### Environmental associations

Associations between genospecies and the environmental factors (geographical area, woodland type, and deer abundance) at each tick collection sites were examined. We chose two broad area categories: the Grampian region, which consists of the Cairngorms, Speyside, Deeside, and Moray in the northeastern quarter of Scotland, and all other sites further west of this region (generally characterized by a warmer and wetter climate than Grampian). Woodlands were categorized as either coniferous or mixed/deciduous. All of the coniferous category were commercial plantations generally consisting of Scots pine *Pinus sylvestris* or spruce *Picea spp*. with larch *Larix decidua*, apart from one conifer site, which was semi-natural old-growth Scots pine. The mixed/deciduous woodlands were semi-natural and consisted primarily of mixed birch *Betula spp*., rowan *Sorbus aucuparia* and sometimes oak *Quercus spp*., and beech *Fagus sylvatica* with occasional Scots pine. The index of abundance of deer was the number of groups of roe and red deer dung pellets counted per 10 m × 1 m transect, averaged for each site (see also Ref. (26)).

To statistically test for associations of area and woodland type (categorical variables) with genospecies we used chi-square tests (a separate test each for area and for woodland type). These reveal differences in habitat and area between the proportions of each genospecies that make up the total number of samples tested at each site. To test for associations of deer abundance (continuous variable) with genospecies, we used a generalized linear mixed model including site as a random effect to account for multiple samples per site. Since the response variable was categorical the default model distribution was multinomial with a cumulative logit link. All statistical tests were conducted in SAS Version 9.3.

## Results

Of the more than 2200 *I. ricinus* tick samples assayed, 124 tested positive to *B. burgdorferi* s.l. and comprised 87 questing nymphs, 25 nymphs removed from birds, and 12 larvae from small mammals (Table 1). Fifty two samples were genotyped at all eight MLST loci and a phylogram of these sequence types was plotted along with most sequence types listed in the MLST database^1^ (Figure S1 in Supplementary Material).

**Table 1:** **Genospecies grouping of Scottish isolates by sample type and the number and type of samples processed using MLST at eight loci**.

Genospecies	Questing nymphs, *N* (%)	Bird nymphs, *N* (%)	Mammal larvae, *N* (%)
*B. afzelii*	42 (48)	0	12 (100)
*B. garinii*	31 (36)	24 (96)	0
*B. burgdorferi* s. s.	6 (7)	0	0
*B. valaisiana*	7 (8)	1 (4)	0
*B. lusitaniae*	0	0	0
*B. afzelii* + *B. garinii*	1 (1)	0	0
No. samples identifiable to genospecies	87	25	12
No. samples analyzed at 8 loci	40 (38 pools of 5 + 2 individuals)	4 individuals	8 pools of 2–28

*Percentages show the proportion of all positive samples represented by the genospecies in question per tick type (questing nymphs, nymphs attached to birds, and larvae attached to small mammals)*.

There was no overlap (sharing) of alleles between any genospecies (either in our data set or in the pubMLST database), implying that samples could be classified into genospecies level without having to genotype at all eight loci, and this also means that identifying mixed genospecies infections is easy. Seventy five samples were genotyped successfully at fewer loci while still allowing the predominant genospecies to be determined. We were not able to amplify all loci for all genospecies. For example, in *B. valaisiana* isolates, the *clpA* locus failed to amplify in six out of nine samples. This is likely attributable to sequence polymorphisms in the PCR primer oligo nucleotide sequences of these strains.

As defined by deep branching of phylogenetic trees, non-sharing of alleles and pair-wise genetic differences above the threshold described in Ref. (20) we identified that, of the total 124 positive tick samples from questing nymphs, nymphs from birds and larvae from small mammals, 55 (44%) contained *B. afzelii*, 56 (45%) contained *B. garinii*, 8 (6%) were *B. valaisiana*, and 6 (5%) were *B. burgdorferi* s.s. Of most importance to Lyme borreliosis risk in humans, out of the 87 questing nymph samples 43 (49%) contained *B. afzelii*, 32 (36%) contained *B. garinii*, 7 (8%) were *B. valaisiana*, and 6 (7%) were *B. burgdorferi* s.s. (Table 1).

### *Borrelia burgdorferi* s.l. within-genospecies diversity

Of the 52 samples typed at eight loci, there were 35 different sequence types, of which 28 were newly described to the MLST database. The most commonly occurring genospecies was *B. afzelii* and its sequence type ST263 accounted for 8/52 samples (15%). The only other sequence types represented more than once in our study were ST287 (*B. afzelii*), ST168 (*B. afzelii*), ST327 (*B. afzelii*), and ST93 (*B. garinii*). All other sequence types were identified from only single samples in this study, although seven had been identified in previous studies^1^: ST24 (*B. burgdorferi* s.s., found in France), ST82 (*B. garinii*, found in France and England), ST86 (*B. garinii*, found in France, England, Latvia, Russia, Austria, and Italy), ST88 (*B. garinii*, found in France and England), ST93 (*B. garinii*, found in France, Italy, and England), ST168 (*B. afzelii* found in Latvia), and ST205 (*B. valaisiana*, found in England).

The phylogenetic tree (Figure S1 in Supplementary Material) shows a greater variety of alleles, and with more branching, in *B. garinii* than in *B. afzelii*. In addition, the extent of nucleotide divergence at the loci was greatest for *B. garinii*, with *B. garinii* samples typically differing by 40–60 bp across the eight loci compared to *B. afzelii* isolates which typically differed by only 1–15 bp.

**Figure 1:**
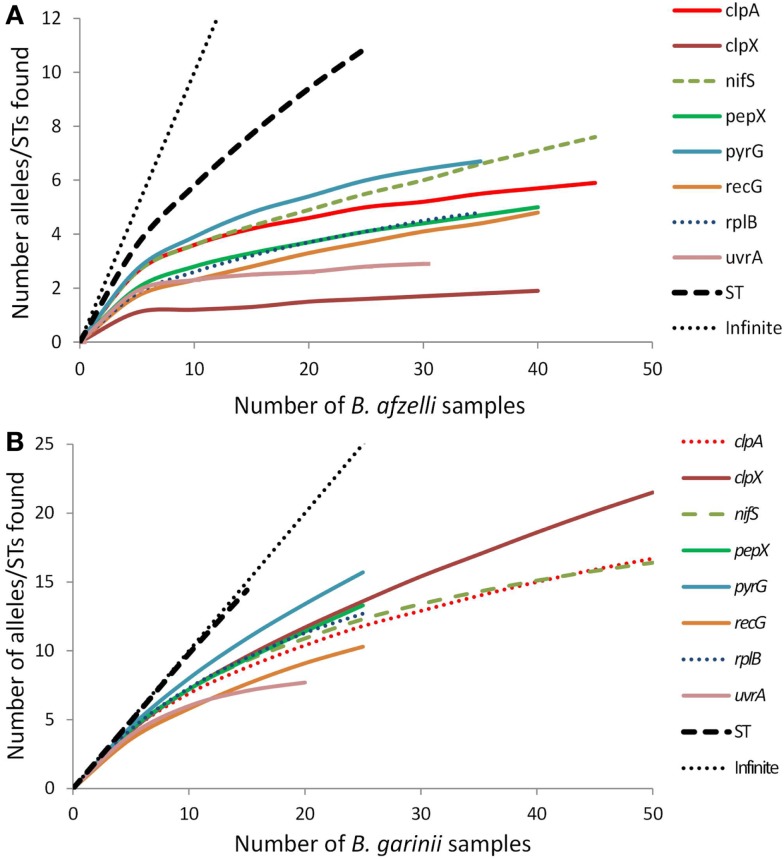
**Rarefaction curves for each of the eight *B*. burgdorferi s.l. loci and sequence types (STs) of (A) B. afzelii samples and (B) B. garinii samples analyzed from Scotland**. Each graph also includes the 45° line (labeled “infinite”) which represents the trajectory that would occur if every new allele or sequence type found was new.

Rarefaction analysis (Figure 1) indicated that for some of the eight loci, especially for *B. afzelii*, we detected most of the alleles in the population in Scotland (the curves plateau) while for other loci, especially for *B. garinii*, there are still several more alleles to be discovered (the curves are still climbing). For both *B. afzelii* and *B. garinii*, the curves for sequence type diversity did not plateau at all; indeed, for *B. garinii* the sequence type curve almost followed the 45^o^ line representing a new discovery for every sample analyzed.

There was a significant positive relationship between genetic and geographic distances for samples within the same genospecies, i.e., samples were more genetically different if they were collected further apart geographically, although the proportion of variation explained by the models (*R*-squared values) was low (*B. afzelii F*_1,407_ = 22.9, *p* < 0.001, *R*^2^ = 51.0%; *B. garinii F*_1,209_ = 15.6, *p* < 0.0001, *R*^2^ = 6.5%). The best fit models were those with simply distance (kilometer); those with (distance)^2^, square root (distance), or log (distance + 1) had poorer fit of the residuals and lower *R*-squared and *F* values.

### Mixed infections

There was evidence of inter-genospecies mixed infections in one sample that clearly contained alleles originating from *B. afzelii* and *B. garinii*. This comprises 1.9% of the 52 samples tested across eight loci or 0.8% of the 124 total samples analyzed (including those successful at fewer than eight loci) and 1.1% of the 87 total samples of questing nymphs.

Close examination of the apparent 14 different sequence types found from the 29 *B. afzelii* samples (Table S1 in Supplementary Material) suggested the possible presence of mixed intra-genospecies infections, deduced as follows. Five sequence types occurred more than once in the population (representing 20 samples) and so are plausibly genuine strains, while five sequence types (from five samples) differed at one of the two loci from the above and their different loci were the result of one or two nucleotide differences from the parental genotype so we considered these also to be plausibly genuine strains (for example ST292 has only one SNP difference in one allele from ST263). However, the remaining four sequence types (ST286, ST288, ST295, and ST326) were represented by only a single sample each and comprised either allele combinations found in other common sequence types (e.g., ST263) or two common sequence types plus a single nucleotide mutation in one of the alleles (Figure S1 in Supplementary Material). It can therefore be speculated that out of 29 *B. afzelii* samples, 25 isolates contained genuine sequence types while four apparent sequence types may actually be examples of intra-genospecies co-infection (i.e., harboring alleles from more than one *B. afzelii* sequence type). If so, this would represent a 14% incidence of intra-genospecies co-infection in the Scottish *B. afzelii* population. However, from only 29 samples, the confidence intervals are wide (upper and lower confidence intervals = 30.6 and 5.5%, respectively).

Homoplasy as a mechanism for the apparent novel sequence types seems unlikely since only two of the four appeared to contain a single nucleotide mutation and in only one of the mixed alleles. There was no evidence that horizontal gene transfer (recombination) is the explanation for these apparent sequence types because no recombination events were detected, either using only our Scottish samples or using a combination of Scottish samples and other sequence types listed in the MSLT database.

### Environmental associations

**Figure 2:**
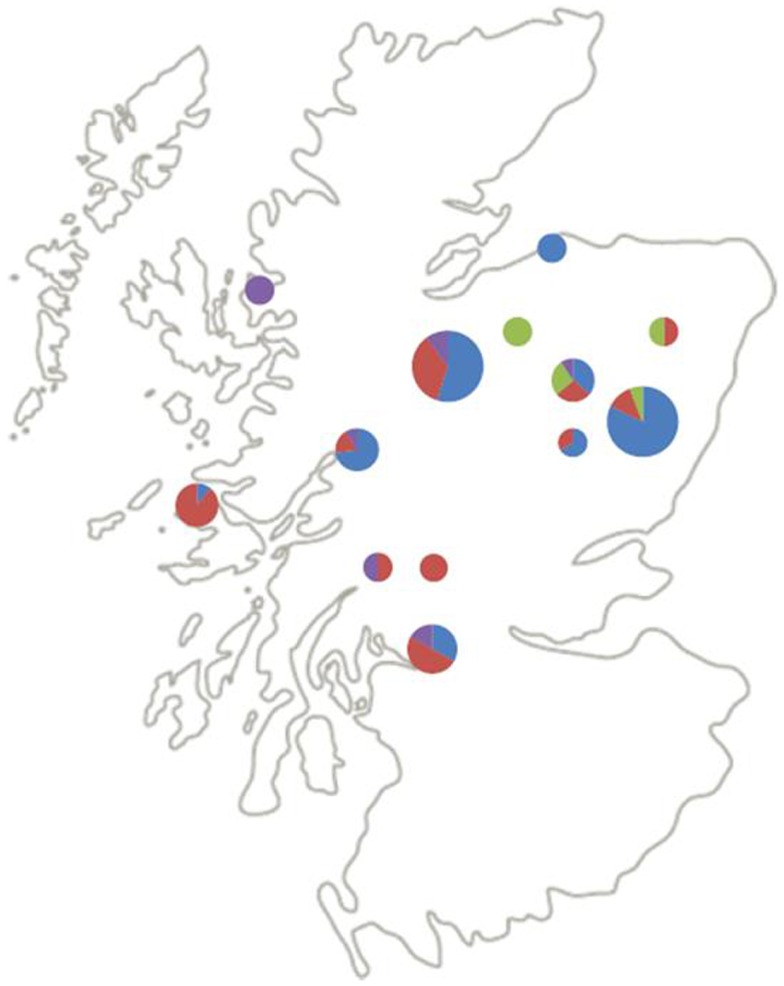
**Distribution and relative abundance of *B*. burgdorferi s.l. genospecies across Scotland**. Blue = *B. afzelii*, red = *B. garinii*, purple = *B. valaisiana*, green = *B. burgdorferi* s.s. The size of the pie charts indicates the number of samples genotyped at that site (1–5, 6–10, and 11–20 samples for small, medium, and large pie charts, respectively).

Figure 2 shows the geographical distribution of the genospecies of the 87 questing nymphs from the 13 (of the original 25) field sites from which positive *B. burgdorferi* s.l. samples were successfully identified to genospecies. Six sites were coniferous and seven semi-natural mixed or deciduous woodlands. *B. afzelii* and *B. garinii*, the most frequent genospecies, seem to be spread over most of Scotland. *B. afzelii* was detected in 7 of the 13 sites and *B. garinii* in 11 of the 13 sites (Table 2). Of the less frequently recorded genospecies, *B. valaisiana* had a broad geographic spread and occurred in 5 of the 13 sites whereas *B. burgdorferi* s.s. was recorded only in 4 sites, all in north-east Scotland (although with only six samples from questing nymphs in this study it may yet be recorded elsewhere if more samples are collected in future studies; Table 2).

**Table 2:** **Genospecies of *B. Burgdorferi* s.l. and sample size of positive samples from questing nymphs at each site**.

Site	Habitat	Deer index	*B. garinii*	*B. afzelii*	*B. valaisiana*	*B. burgdorferi* s.s.	Total *B. burgdorferi* s.l.
QC	Conifer	0.94	3 (27%)	4 (36%)	1 (9%)	3 (27%)	11
GM	Conifer	0	2 (50%)	0 (0%)	2 (50%)	0 (0%)	4
BM	Conifer	0.61	1 (33%)	2 (67%)	0 (0%)	0 (0%)	3
CB	Conifer	0.35	0 (0%)	3 (100%)	0 (0%)	0 (0%)	3
IR	Conifer	0.08	0 (0%)	0 (0%)	0 (0%)	2 (100%)	2
AP	Conifer	0	0 (0%)	0 (0%)	1 (100%)	0 (0%)	1
DR	Mixed	0.01	7 (35%)	11 (55%)	2 (10%)	0 (0%)	20
FZ	Mixed	0	2 (12%)	14 (82%)	0 (0%)	1 (6%)	17
LA	Mixed	0	2 (18%)	8 (73%)	1 (9%)	0 (0%)	11
DV	Mixed	0.07	8 (89%)	1 (11%)	0 (0%)	0 (0%)	9
SH	Mixed	0	3 (50%)	2 (33%)	1 (17%)	0 (0%)	6
TB	Mixed	0.11	3 (100%)	0 (0%)	0 (0%)	0 (0%)	3
LV	Mixed	0.06	1 (50%)	0 (0%)	0 (0%)	1 (50%)	2
All			32 (35%)	45 (49%)	8 (9%)	7 (8%)	92

*Deer index is the number of pellet groups per 10 m × 1 m transect averaged per site*.

**Figure 3:**
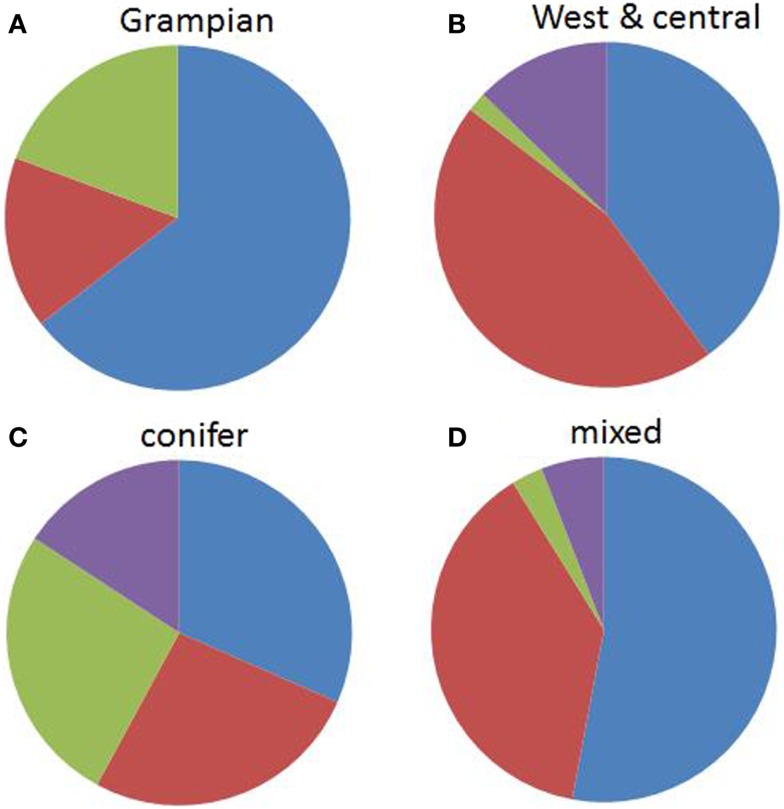
**The proportions of *Borrelia burgdorferi* s.l. genospecies by area:** **(A)** west and central Scotland and **(B)** the Grampian Region of Scotland (the Northeast and the Cairngorms) and by habitat: **(C)** conifer forest and **(D)** mixed/deciduous woodlands, averaged over 13 (6 coniferous, 7 mixed/deciduous) sites across Scotland. Blue = *B. afzelii*, red = *B. garinii*, purple = *B. valaisiana*, and green = *B. burgdorferi* s.s.

There was a significant difference between genospecies and the two broad area categories (*χ*^2^ = 18.8, df = 3, *p* = 0.0003). *B. garinii* and *B. valaisiana* comprised the highest proportions of samples in the western half of Scotland while *B. burgdorferi* s.s. was most associated with the Grampian region (Figure 3). There was also a significant difference between genospecies and woodland type (*χ*^2^ = 13.9, df = 3, *p* = 0.0031). *B. afzelii* and *B. garinii* were more likely to be found in mixed/deciduous than in conifer forests and *B. burgdorferi* s.s., while occurring at only low prevalences, had a tendency to be found more frequently in coniferous forest (Figure 3). There was no evidence for a significant association between genospecies and the deer abundance at each site (*F*_1,72_ = 0.53, *p* = 0.468).

## Discussion

The overall *B. burgdorferi* s.l. prevalence of 5.6% (range 1–14%) in questing nymphs [see also Ref. (26)] is similar to that found in a previous UK study (15) that found 5 and 8% prevalence in 2006–2007 and 2008–2009, respectively (range 0–12%) in ticks collected from sites mainly in England. This is a lower prevalence than that found in many countries in continental Europe [e.g., Ref. (3, 15)].

Of the *B. burgdorferi* s.l. positive questing nymphs, 49% were *B. afzelii*, 36% were *B. garinii*, 8% were *B. valaisiana*, and 7% were *B. burgdorferi* s.s. The finding that the most common genospecies in Scotland is *B. afzelii* [see also Ref. (24) which found that almost half of its 12 positive *I. ricinus* from the Scottish highlands carried *B. afzelii*] while *B. valaisiana* is relatively rare is interesting, because a quite different pattern has been found in England where *B. afzelii* is less common while *B. garinii* and *B. valaisiana* (the two bird-associated genospecies) seem to be predominant [e.g., Ref. (38)], although Ref. (15) found *B. afzelii* at around half of their English sites. The reasons for any difference in genospecies predominance between Scotland and the rest of the British Isles warrants further research. The abundance of *B. afzelii* in Scotland is more similar to the situation in continental Europe where it is also common and often the dominant genospecies [e.g., Ref. (3, 39, 40)].

Corroborating current knowledge of genospecies specificity [e.g., Ref. (38)], we found that the sole genospecies found in larval ticks removed from small mammals (wood mice and bank voles) was *B. afzelii* while, as expected, *B. garinii* was the dominant genospecies found in nymphs removed from passerine birds (and one *B. valaisiana* sample from a bird).

### Within-genospecies diversity

As we predicted from the concept that the spatial distribution of genetic relatedness is driven by local host movements, for both *B. afzelii* and *B. garinii*, we found a positive relationship between geographical distance (kilometer separating field sites) and genetic distance (single nucleotide polymorphism differences) between pairs of samples within genospecies, i.e., alleles and sequence types are more different the further apart they are geographically. However, the most abundant strain type (*B. afzelii* ST263) occurs widely across Scotland (up to 110 km in this study), and could perhaps, therefore, be an ancestral strain. *B. garinii* was considerably more genetically diverse than *B. afzelii* (that was dominated by ST263). This is consistent with our predictions based on higher number of bird species than small mammal species and the higher frequency of non-local alleles being brought into Scotland from other countries by migrating birds. We found that all (apart from one, previously identified from Germany) of the *B. afzelii* sequence types we identified from Scotland were novel. This helps corroborate the proposal of Ref. (15) (stemming from previous limited data on Scottish *Borrelia burgdorferi* s.l.) that there should be a clear distinction between English and Scottish *B. afzelii* due to limited movement of small mammals between England and Scotland [see Ref. (41)]. In contrast to *B. afzelii*, several *B. garinii* sequence types that we identified in Scotland had previously been identified from France and England, suggestive of greater host (bird) movement between these countries. A high degree of diversity in *B. garinii* samples has also been noted in Ref. (42), among others, who attributed this to their bird host’s large migration ranges.

### Mixed infections

The identification of mixed samples can be influenced by the extent of horizontal gene transfer events, which will decrease the observed clonality of the species. In the case of *B. burgdorferi* s.l., however, there is no evidence to support horizontal gene transfer of chromosomal genes and, certainly between genospecies, the deep branching, and absence of pan-alleles argues against horizontal gene transfer. We found only one sample clearly exhibiting alleles from two genospecies, which is most likely to be a result of mixed-genospecies infections within a tick. This is a frequency of 1–2% (depending on whether the total number of 124 samples is used or only those 52 samples analyzed at all eight loci or including only questing nymphs). This is much lower than many other areas: a meta-analysis in Ref. (3) of over 100 articles citing the infection prevalences in 112,579 questing ticks from 24 countries across Europe found that the occurrence of inter-genospecies co-infection in Ixodid nymphs was, overall, 12.1%, while as many as 64% of questing nymphs were co-infected in Denmark (43).

Assuming that transovarial transmission of *B. burgdorferi* is absent or rare such that unfed larvae are uninfected (16, 44) outlined three potential mechanisms for an unfed nymph to be co-infected with more than one genospecies: (i) through transmission by co-feeding (feeding in close proximity to a tick infected with a different genospecies to that in the host); (ii) through being unable to complete a full blood meal from one host (e.g., dislodged before repletion) and so feeding from a second host containing a different *B. burgdorferi* s.l. genospecies; or (iii) through feeding from a single host that carried more than one genospecies, perhaps because of a compromised immune system. Most mixed infections in Europe are *B. garinii* mixed with *B. valaisiana* (3) (i.e., the two genospecies associated with birds). It is noteworthy, therefore, that our two mixed genospecies samples contained alleles from *B. afzelii* and *B. garinii*, the small mammal- and the bird-associated genospecies respectively. Therefore, it is unlikely that our mixed infection resulted from a host infected with both genospecies. However, it could have resulted from the tick having an incomplete feed on a *B. afzelii* infected small mammal followed by a further feed on a *B. garinii* infected bird (or *vice versa*). It could also have potentially resulted from co-feeding: the tick attached to a bird or small mammal infected with one pathogen while feeding in close proximity to a nymph that was infected with the second pathogen.

While mixed infections of different genospecies are relatively easy to identify in samples and are now well documented [e.g., Ref. (3, 15)], it may be more likely for a tick to be co-infected with different strains of the same genospecies (each with a different sequence type), although it is much less easy to identify. This phenomenon has been previously reported: 39% of adult *Ixodes scapularis* ticks from North America were infected with more than one genotype of *B. burgdorferi* s.s. (45). From close examination of the novel sequence types we found, we estimated that the incidence of intra-genospecies co-infection is around 14% in the Scottish *B. afzelii* population. We also considered the alternative explanations for these four apparent sequence types. The allele combinations ruled out vertical inheritance. Homoplasy is unlikely since several of the shared alleles harbor more than one single nucleotide polymorphism compared to the putative ancestral strain in several cases. In addition, it has been reported as an unlikely event in the *B. burgdorferi* s.l. genome and nearly non-existent in the chromosome [reviewed in Ref. (46)]. At an environmental scale, there is no evidence of horizontal gene transfer in the IGS locus, but the *ospC* locus showed evidence of intragenic recombination (47). It would appear that recombination events are possible within the *B. burgdorferi* s.l. genome, but they are rare and limited to particular genes (e.g., *ospC*). *ospC* may be subject to recombination in a way that other genes are not due to its role in the immune system (unlike the selectively neutral housekeeping genes of MLST) (48).

Our finding of commonly occurring mixed within-genospecies co-infections is important also because it suggests that the MLST database may inadvertently contain examples of mixed intra-genospecies co-infection. Detecting and excluding these samples could be overcome only by culturing colonies from ticks and selecting individual colonies or by using detection methods, which can pick up multiple genospecies in a sample. Unless samples in the MLST database contain sequence types from individual colony cultures (the majority are not, as they are from field collected whole ticks), then the database may contain many of these intra-genospecies mixed sequence types and should therefore be treated with caution.

More work is required to examine this phenomenon of mixed infections, for example by the cultural separation of samples with mixed chromatograms or forensic methods such as designing PCR probes to identify individual genospecies or even specific alleles. This may also help answer whether intra-genospecies co-infection is more common within certain genospecies. It perhaps would be more likely for intra-genospecies co-infection to occur in *B. garinii*, as there is more diversity of allele numbers.

### Environmental associations

*Borrelia afzelii* was found relatively evenly throughout Scotland whereas *B. burgdorferi* s.s. was found only around the Grampian region of north east Scotland (albeit detected in only nine samples). *B. garinii* and *B. valaisiana* were widely spread across Scotland although, statistically, they occurred at higher proportions in the warmer and wetter western areas of Scotland. These distributional differences are likely to be associated with differences in relative host abundances, which are generally driven by habitat which in turn is affected by both climate and anthropogenic land management goals. Four of the six (67%) Grampian sites were coniferous, compared with two of the seven (29%) western sites but within each of our two very basic woodland categories are many sub-categories of habitat such as lowland deciduous, upland birch, Atlantic oak, juniper scrub, ancient pine, and commercial plantation. While the number of sites we originally sampled was 25, many more would be needed to statistically test genospecies associations with finer habitat categories. However, using our two basic woodland types, *B. garinii* and *B. afzelii* more likely to be found in deciduous forests while higher proportions of *B. burgdorferi* s.s. were found in coniferous forest. This is likely to reflect the differences in relative abundance of host types between habitats: *B. garinii* and *B. afzelii* are associated with birds and small mammals, respectively, and semi-natural mixed or deciduous woodlands are generally associated with higher abundance and biodiversity of both birds and small mammals than are conifer plantations. However, it is unclear why *B. burgdorferi* s.s. should occur more frequently in coniferous woodlands, since previous studies suggest that the key reservoir hosts for *B. burgdorferi* s.s. are, as for *B. afzelii*, small mammals [e.g., Ref. (49, 50)]. Given the low number of *B. burgdorferi* s.s. positive samples we found in Scotland, this could be a statistical artifact, but further work is required to identify the key reservoir host for *B. burgdorferi* s.s. in Scotland and its relative abundance between habitats. Given that the spatial distribution of *B. burgdorferi* s.s. seems to be restricted to the Grampian Region and Speyside, and there may be an association with coniferous forests, we can speculate that red squirrels *Sciurus vulgaris* (that are also more abundant in conifer forests in this region than many other parts of Scotland) could be important *B. burgdorferi* s.s. in Scotland. Indeed, *B. burgdorferi* s.s. is prevalent in red squirrels in Switzerland and red squirrels can transmit *B. burgdorferi* s.s. to feeding ticks (51). Similarly, associations have been found between *B. burgdorferi* s.s. and western gray squirrels *Sciurus griseus* in California (52).

## Conclusion

This large-scale intensive analysis of more than 2000 *I. ricinus* tick samples from over 1200 10 m × 1 m transect surveys, and from birds and small mammals, at 25 sites has provided the first comprehensive analysis of the *B. burgdorferi* s.l. genospecies present in Scotland. That the most prevalent genospecies was *B. afzelii* was surprising as it has been postulated to be rare in the United Kingdom (2, 15). We found much lower inter-genospecies co-infections (1%) than found in other countries but, importantly, we found frequent intra-genospecies co-infections (14% of *B. afzelii*), suggesting co-feeding ticks, ticks feeding on multiple hosts, or multiple infections within hosts. Our findings that genetic and geographic distances are positively correlated and the differences in intra-genospecies genetic diversity can help us understand how, and from where, each pathogen spreads spatially over time. We speculate that red squirrels may be an important reservoir host for *B. burgdorferi* s.s. in northeastern Scotland, from circumstantial evidence of its regional and habitat associations, as well as previous evidence from Switzerland. The association between some genospecies and geographic area could be useful to practitioners in diagnostics, since each genospecies varies in the symptoms caused, especially if future work can determine the pathogenicity of different local strains. By examining the spatial patterns of genospecies and strain types in many countries, and linking this to their pathogenicity, it may become possible to understand the heterogeneous spatial distributions of genospecies, disease risk, and patient symptoms across more globally.

## Conflict of Interest Statement

The authors declare that the research was conducted in the absence of any commercial or financial relationships that could be construed as a potential conflict of interest.

## Supplementary Material

The Supplementary Material for this article can be found online at http://www.frontiersin.org/Journal/10.3389/fpubh.2014.00129/abstract

## Footnotes

^1^www.mlst.net
