# Supplementary material for: The Heterogeneity, Distribution, and Environmental Associations of Borrelia burgdorferi Sensu Lato, the Agent of Lyme Borreliosis, in Scotland
Source: Front Public Health. 2014 Aug 28;2:129. doi: 10.3389/fpubh.2014.00129 (PMC4147938; doi:10.3389/fpubh.2014.00129)
Supplement: Supplementary file 1 [file Presentation1.PDF]

Supplementary material

Table S1. Allele table of all samples processed at eight loci. New alleles and sequence types (STs) are highlighted in bold. *B.b.s.s* means *B. burgdorferi* s.s.

Sample	Genospecies	*clpA*	*clpX*	*nifS*	*pepX*	*pyrG*	*recG*	*rplB*	*uvrA*	ST
DR37	*B. afzelii*	37	24	24	31	22	29	23	**99**	**292**
M114	*B. afzelii*	37	24	24	31	22	29	23	28	**263**
M19	*B. afzelii*	37	24	24	31	22	29	23	28	**263**
M111	*B. afzelii*	37	24	24	31	22	29	23	28	**263**
M92	*B. afzelii*	37	24	24	31	22	29	23	28	**263**
M95	*B. afzelii*	37	24	24	31	22	29	23	28	**263**
LA24	*B. afzelii*	37	24	24	31	22	29	23	28	**263**
FZ5	*B. afzelii*	37	24	24	31	22	29	23	28	**263**
LA22	*B. afzelii*	37	24	24	31	22	**109**	23	28	**294**
LA25	*B. afzelii*	37	24	24	31	22	29	23	28	**263**
DR18	*B. afzelii*	37	24	24	31	22	29	23	28	**263**
M103	*B. afzelii*	37	24	24	31	22	29	23	28	**263**
FZ10	*B. afzelii*	37	24	24	31	22	29	23	28	**263**
DR38	*B. afzelii*	37	24	24	31	22	29	23	28	**263**
FZ4	*B. afzelii*	37	24	24	31	22	29	23	28	**263**
FZ48	*B. afzelii*	36	24	24	31	22	29	**90**	28	**287**
QC24	*B. afzelii*	36	24	24	31	22	29	**90**	28	**287**
M39	*B. afzelii*	36	24	**89**	31	22	29	23	28	**288**
DR10	*B. afzelii*	37	24	24	31	22	29	**90**	28	**291**
FZ49	*B. afzelii*	37	24	24	31	22	29	**91**	28	**293**
QC28	*B. afzelii*	37	24	23	31	22	29	23	28	**289**
M58	*B. afzelii*	36	24	23	32	**96**	29	23	28	**286**
LA33	*B. afzelii*	36	24	23	31	85	27	23	29	168
LA45	*B. afzelii*	36	24	23	31	85	27	23	29	168
BM40	*B. afzelii*	109	24	25	86	24	**110**	23	29	**326**
SH21	*B. afzelii*	38	24	25	32	24	29	24	28	**295**
QC6	*B. afzelii*	109	24	25	86	**107**	29	23	29	**327**
FZ25	*B. afzelii*	109	24	25	86	**107**	29	23	29	**327**
FZ28	*B. afzelii*	109	24	25	86	**107**	29	23	29	**327**
QC27	*B.b.s.s.*	14	1	11	1	1	11	1	10	**284**
FZ3	*B.b.s.s*	15	9	12	8	1	11	11	16	**285**
LV4aM	*B.b.s.s*	15	9	12	8	1	11	8	16	24
GM27	*B. valaisiana*	110	39	36	45	38	44	35	40	205
FZ47	*B. garinii*	40	25	26	36	27	34	25	31	82
b90	*B. garinii*	43	28	30	39	30	36	**92**	34	**299**
GM15	*B. garinii*	43	28	30	39	30	36	28	34	88
LV5M	*B. garinii*	43	28	**92**	90	30	36	28	34	**303**
b26	*B. garinii*	43	27	30	38	**108**	36	28	34	**298**
DV46	*B. garinii*	46	74	**90**	**110**	**109**	54	29	35	**312**
DR46	*B. garinii*	42	77	33	44	29	36	27	33	**297**
LA34	*B. garinii*	99	77	**81**	91	88	**113**	**82**	33	**325**
TS1	*B. garinii*	42	27	29	**113**	**110**	36	42	33	**296**
TS2	*B. garinii*	42	27	29	38	29	36	27	33	86
DR49	*B. garinii*	95	**90**	34	**111**	89	78	77	85	**323**

LA9	*B. garinii*	95	**90**	**91**	96	89	**111**	77	85	**324**
b66	*B. garinii*	45	33	34	36	36	38	30	38	93
QC14	*B. garinii*	45	33	34	36	36	38	30	38	93
DV12	*B. garinii*	45	33	78	36	81	38	**93**	38	**304**
b34	*B. garinii*	46	30	**91**	**112**	**111**	**111**	30	36	**310**
DV23	*B. garinii*	47	27	33	42	29	**112**	32	33	**319**
DV13	*B. garinii*	47	27	33	42	91	**111**	32	36	**320**
DV28	*B. garinii*	47	**89**	33	42	91	36	32	36	**321**

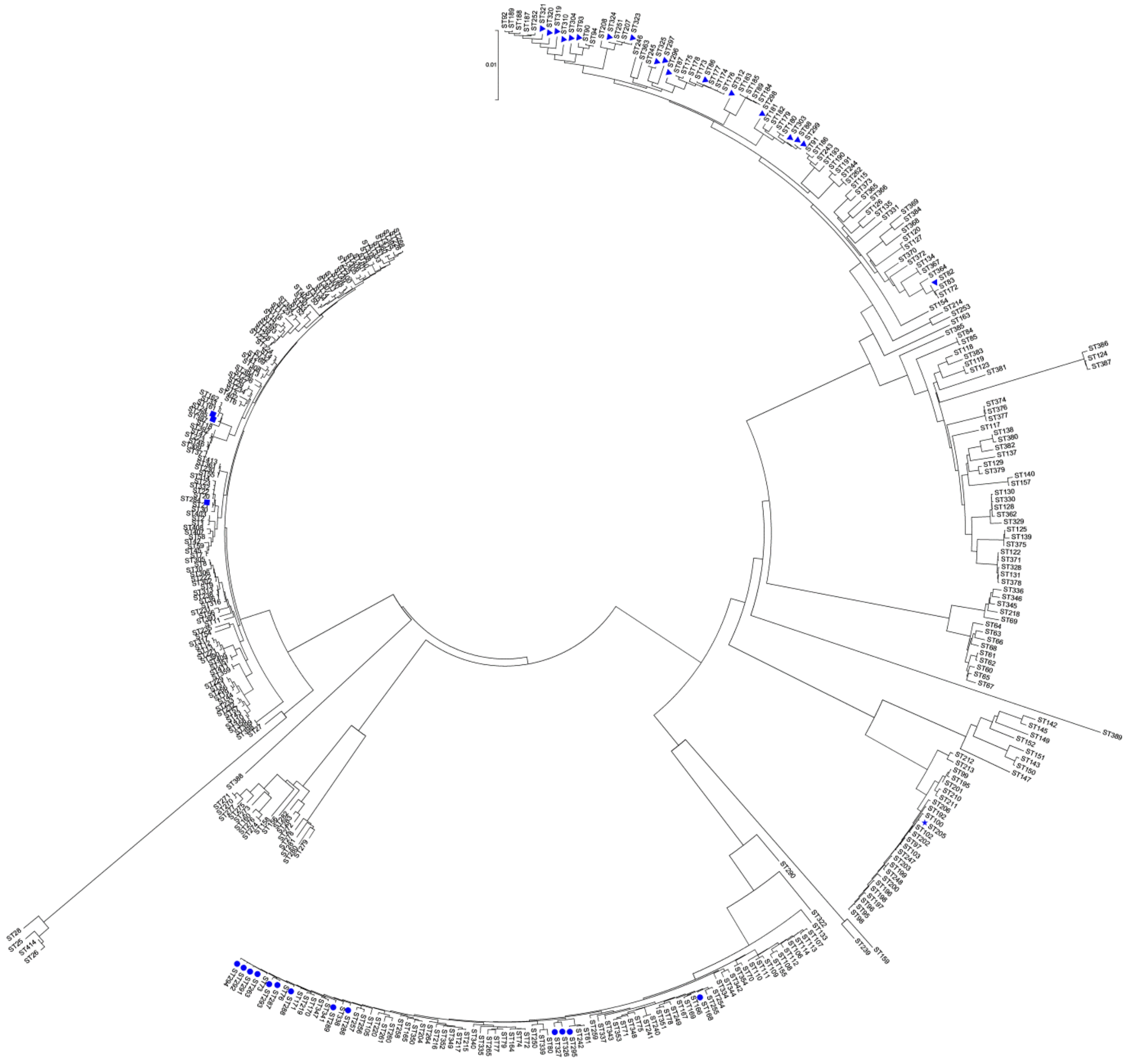

Figure S1. Neighbour joining tree of *B. burgdorferi* s.l. strains based on concatenated DNA sequences of the eight MLST loci of isolates from this study plus others selected from elsewhere in the world (from pubMLST). Strains from this study are identified by blue triangles (*B. garinii*), blue circles (*B. afzelii*), blue star (*B. valaisiana*) or blue squares (*B. burgdorferi* s.s.). The scale represents nucleotide differences.
